# The tumour response of postmenopausal hormone receptor-positive breast cancers undergoing different types of neoadjuvant therapy: a meta-analysis

**DOI:** 10.1186/s12905-020-0879-y

**Published:** 2020-01-31

**Authors:** Yaling Wang, Lin He, Yuhua Song, Qian Wu, Haiji Wang, Biyuan Zhang, Xuezhen Ma

**Affiliations:** 1 Department of Oncology, The Second Affiliated Hospital of Medical College of Qingdao University, No. 127 Siliunan Road, City North District, Qingdao, Shandong Province China; 2 Breast Center B ward, The Affiliated Hospital of Qingdao University, Qingdao, Shandong Province China; 3 Department of Radiotherapy, The Affiliated Hospital of Qingdao University, Qingdao, Shandong Province China; 4 Department of Oncology, Qingdao Central Hospital, No. 127 Siliunan Road, City North District, Qingdao, Shandong Province China

**Keywords:** Neoadjuvant endocrine therapy, Neoadjuvant chemotherapy, Neoadjuvant chemoendocrine therapy, Tumour response, Breast cancer

## Abstract

**Background:**

To investigate the efficacy of neoadjuvant chemotherapy (NCT), neoadjuvant endocrine therapy (NET) and neoadjuvant chemoendocrine therapy (NCET) on the tumour response, including pathological complete response (pCR) rate and overall response rate (ORR), in postmenopausal women with hormone receptor (HR)-positive breast cancer.

**Methods:**

Based on a PRISMA-IPD statement, the PubMed, Embase and Cochrane Library databases were used to identify eligible trials published from inception to 7 May 2019. Pooled odds ratio (OR) with 95% confidential interval (CI) was calculated to assess the pCR rate and ORR of tumours among those three treatments via fixed- or random-effect Mantel-Haenszel models in terms of a Heterogeneity Chi^2^ test with a significant level of *p* < 0.1. All statistical tests were performed by the software of StataSE, version 12.0.

**Results:**

The analysed data consisted of 10 eligible clinical trials with 971 unique HR-positive breast cancer patients. The pooled results indicated that the pCR rate of those patients undergoing NET was significantly lower than those undergoing NCT (pooled OR, 0.48; 95% CI, 0.26–0.90), whereas the difference of ORR between both therapies was not statistically significant (pooled OR, 1.05; 95% CI, 0.73–1.52). The combined paradigm of NCET compared with the monotherapy of NET or NCT did not present a significantly improved pCR rate or ORR (pooled OR, 2.61; 95% CI, 0.94–7.25; and 2.25; 95% CI, 0.39–13.05; respectively).

**Conclusion:**

Postmenopausal HR-positive breast cancer patients after NCT may have better tumour response than those after NET, while those undergoing NCET may not manifest the apparently improved clinical efficacies compared to those receiving monotherapy.

## Background

The initial clinical experience of neoadjuvant therapy in breast cancer was to use it in locally advanced, inoperable tumours, showing excellent tumour response and ameliorative local control [1], as well as equivalent overall survival (OS) and disease-free survival (DFS) compared with adjuvant therapy [2, 3]. Neoadjuvant therapy for hormone receptor (HR)-positive breast cancer includes neoadjuvant chemotherapy (NCT), neoadjuvant endocrinotherapy (NET) and neoadjuvant chemoendocrine therapy (NCET). After these treatments, unresectable tumours can be made amenable to mastectomy, and the initially operable ones in requirement of mastectomy may be operated on in order to avoid sacrificing the breast via lumpectomy, thereby greatly extending long-term survival and improving the quality of life.

The National Surgical Adjuvant Breast and Bowel Project (NSABP) B-18 study found a positive correlation between higher pathological complete response (pCR) rate and improved relapse-free survival in HR-positive breast cancer patients who underwent adjuvant or neoadjuvant chemotherapy [4]. Similarly, a multicenter phase II study by the Japan Breast Cancer Research Group (JBCRG) demonstrated that the achievement of pCR in HR-positive breast cancer women who received NCT predicted favourable DFS [5]. These results suggest that pCR, overall response rate (ORR) and other biomarkers can be used as valid surrogate endpoints to predict long-term survival in patients after neoadjuvant treatment.

Some studies collectively confirmed that the pCR rate of HR-positive breast tumours following NCT was lower than that of HR-negative breast cancers [5–7]. Moreover, the majority of breast carcinoma patients with HR-positive tumours are postmenopausal and elderly and, therefore, have difficulty tolerating the adverse effects of NCT. These phenomena predispose them to choose NET, which has lower toxicity and similar clinical efficacy compared with NCT [8, 9]. However, a poor clinical response rate to NET is shown in highly aggressive (Ki67 > 10%) HR-positive breast tumours when compared to NCT [9], suggesting that these two treatment strategies are not equally effective in certain cases.

NCET, which concurrently applies NET in combination with NCT has attained promising antitumour activity in animal models and human bodies with breast cancer, resulting in more shrinkage of tumour volume and reduction of tumour cell proliferation compared to NET [10–12]. Many clinical studies have investigated the ORR of both modalities in treating HR-positive breast tumours but have reported somewhat different findings [11, 13, 14]. Herein, we set out to investigate these three neoadjuvant therapy scenarios to determine which one achieved the greatest tumour response rate in treating postmenopausal HR-positive breast carcinoma. Four comparison groups were designed, including the pCR rate and ORR of NET vs NCT and those of NCET vs NET or NCT alone.

## Methods

### Search strategy

According to the PRISMA-IPD Statement [15], electronic searching was performed in databases including PubMed, Cochrane Library and Embase using the following retrieval strategy: ((neoadjuvant endocrine therapy) OR (neoadjuvant endocrine treatment) OR (neoadjuvant endocrinotherapy) OR (neoadjuvant chemotherapy) OR (neoadjuvant therapy) OR (neoadjuvant chemoendocrine therapy) OR (neoadjuvant chemo-endocrine therapy) OR (neoadjuvant chemoendocrine treatment) OR (neoadjuvant chemo-endocrine treatment)) AND ((breast neoplasms) OR (breast cancer) OR (breast tumour) OR (breast tumour) OR (breast carcinoma)) AND (post-menopausal OR postmenopausal) AND ((hormone receptor positive) OR (oestrogen receptor positive) OR (estrogen receptor positive) OR (hormone receptor rich) OR (oestrogen receptor rich) OR (estrogen receptor rich) OR Luminal) AND (pCR OR (pathological complete response) OR ORR OR (overall response) OR (clinical response) OR (tumour response) OR (tumour response)). No restrictions were required during retrieval. Citations were terminated searching as of 7 May 2019.

### Inclusion criteria

- English publication;
- Retrospective or prospective clinical trials;
- Postmenopausal women with HR-positive breast cancer;
- Publication reported the comparison of tumour response rate between either NET with NCT or NCET with NET or NCT alone. Tumour response rate involved pCR rate and ORR. The definition of pCR was that no malignant tumour cells were pathologically detected at the original tumour site. ORR referred to the merger value of complete response rate and partial response rate that were assessed by solid tumours after neoadjuvant therapy. The tumour response was evaluated in light of Response Evaluation Criteria in Solid Tumours, version 1.1 (RECIST v1.1) or World Health Organization (WHO) assessment criteria.

### Exclusion criteria

- Trials published in non–English;
- Article type: review, case report, study protocol and conference paper;
- Other details that did not meet the inclusion criteria.

Two reviewers independently screened the titles and abstracts of citations and scrutinized the full text of all potential studies. Only the satisfactory ones were retained. If there were some inconsistencies, they were figured out by discussion.

### Data abstraction

The following information was abstracted from included studies by two co-authors who utilized the software of Excel version 2016: first author, publication year, study duration, original nation, median age, median follow-up, detailed regimen of treatment, total sample size, the event number of pCR and clinical response after individual therapy. If some unconformities existed, they were resolved by the third co-author. The datasets used and/or analyzed during the current study are available from the corresponding author on reasonable request.

### Statistical analysis

The comparisons of pCR and ORR between the study cohort and the control cohort were presented by the value of pooled odds ratio (OR). The crude ORs with 95% confidence intervals (CIs) in all included studies were independently calculated then pooled together. If the publication did not provide the number of events, the calculation was in terms of the endpoint percentage or other information. The heterogeneity among different trials was assessed by Heterogeneity Chi^2^ test with a significance level of *p* < 0.1 [16]. When the heterogeneity test appeared to indicate no statistical significance (*p* ≥ 0.1), a fixed-effect Mantel-Haenszel model was utilized to pool the data; if not, a random-effect Mantel-Haenszel model was used [16]. The publication bias was investigated by Egger’s test with a significance level of *p* < 0.05. The quality of all RCTs was evaluated in terms of the instrument presented by Jadad and colleagues (Additional file 1: Table S1, *page 1*) [17]. All statistical tests were analysed using the software StataSE version 12.0 (StataCorp LP, College Station, TX, USA).

## Results

### Search results

**Fig. 1:**
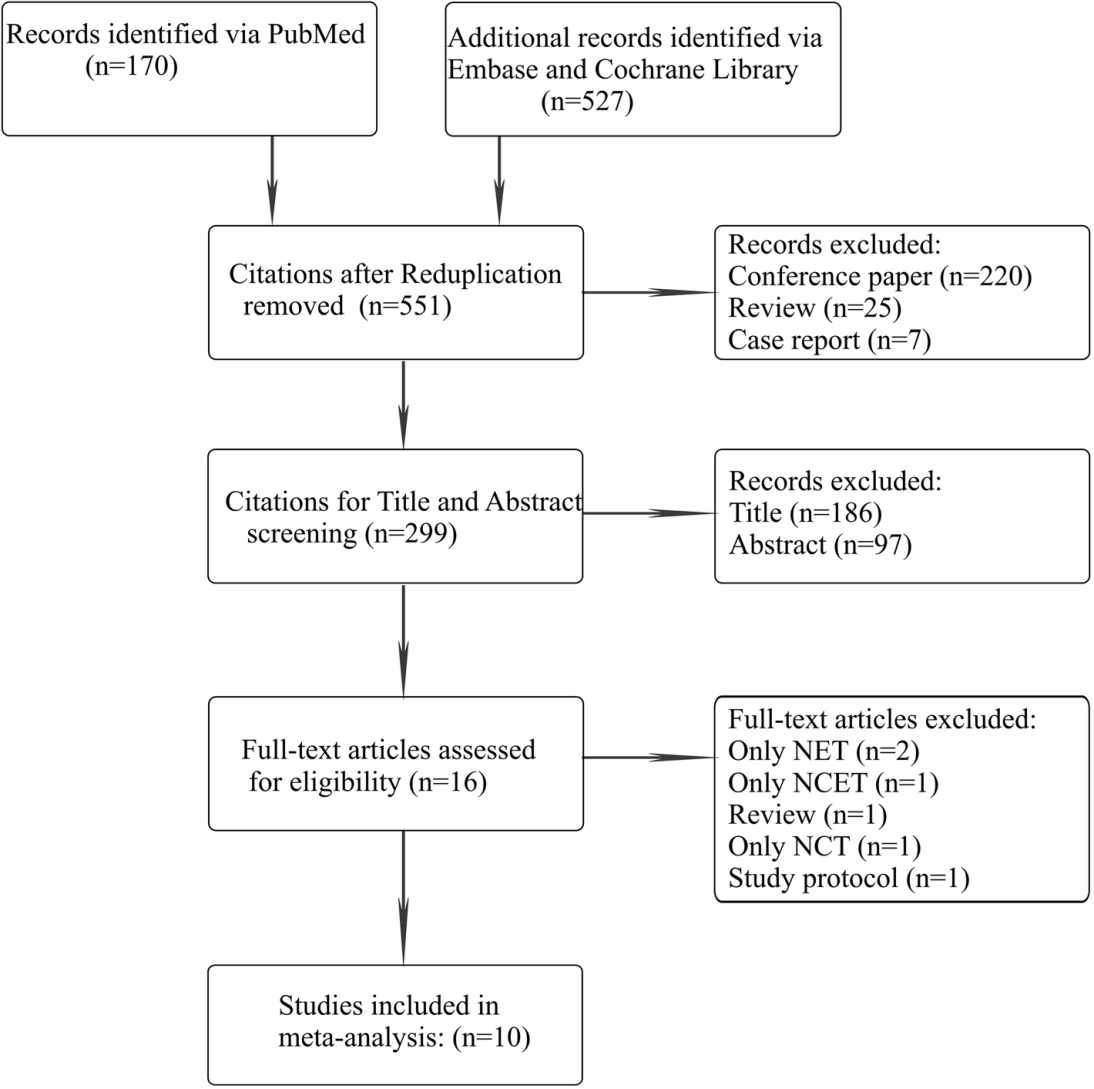
The PRISMA flow diagram of the inclusion process. Abbreviations; NCET, neoadjuvant chemoendocrine therapy; NCT, neoadjuvant chemotherapy

Following systematic retrieval, a total of 697 potential citations were identified. Citations that were duplicates (*n* = 146) or categorized as review (*n* = 25), case report (*n* = 7) and conference papers (*n* = 220) were excluded. The remaining 299 citations were entered into title and abstract screening, and 283 citations were removed. Therefore, sixteen studies were left for full-text scrutinizing, and 6 of them were omitted because of only NET (*n* = 2), only NCET (*n* = 1), only NCT (*n* = 1), review (*n* = 1) or study protocol (*n* = 1). Ultimately, ten eligible clinical trials with 971 unique HR-positive breast cancer patients met the inclusion criteria [8, 11, 13, 14, 18–23]. The PRISMA flow diagram of the inclusion process was outlined in Fig. 1**.**

### Characteristics of eligible studies

Of the included trials, the publication year ranged from 2007 to 2018; four recorded the types of surgery after neoadjuvant treatment, in which only one assessed the nodes management; three reported the toxicity after treatments that focused on NCET vs NET or NCT monotherapy; the baseline clinical stages were mainly in T1/2 (*n* = 3), T3/4 (*n* = 3) and unknown (*n* = 4); the lengths of neoadjuvant treatment were different, ranging from 9 to 24 weeks; the HER2 status was negative (*n* = 4), or negative/positive (*n* = 5) or undescribed (*n* = 1), however, the number of patients with HER2-positive disease was scarce, with a total number of 29 (ranged from 2 to 18). Table 1 also represented other detailed characteristics of included studies. The treatment regimens for NET, NCT and NCET strategies were outlined in Table 2.

**Table 1:** The details of included studies

Characteristic	Studies, No. (%) (*N* = 10)	Postmenopausal HR+ Breast Cancer Patients, No. (%) (*N* = 971)
Study type		
Randomized clinical trial	7 (70.0)	676 (69.6)
Retrospective	2 (20.0)	239 (24.6)
Prospective	1 (10.0)	56 (5.8)
Publication date, median (range), y	2015 (2012–2018)	
Follow-up, median (range), mo^a^	48.1 (17.3–74)	
Mean age, median (range), y^a^	60.9 (49–70)	
Original nation		
the United States	3 (30.0)	460 (47.4)
Korea	1 (10.0)	25 (2.6)
Japan	3 (30.0)	141 (14.5)
the United Kingdom	1 (10.0)	44 (4.5)
Russia	1 (10.0)	239 (24.6)
Iran	1 (10.0)	62 (6.4)
Assessment of type of surgery		
Yes	4 (40.0)	378 (38.9)
No	6 (60.0)	593 (61.1)
Nodes management		
Yes	1 (10.0)	221 (22.8)
No	9 (90.0)	750 (77.2)
Toxicity		
NET vs NCT	0 (0.0)	0 (0.0)
NECT vs NET	2 (20.0)	113 (11.6)
NECT vs NCT	1 (10.0)	62 (6.4)
Not assessed	7 (70.0)	796 (82.0)
Assessment criteria of tumour response		
RECIST v1.1	5 (50.0)	244 (25.1)
WHO	1 (10.0)	239 (24.6)
Not assessed	4 (40.0)	488 (50.3)
Mainly baseline clinical stage		
T1/2	3 (30.0)	183 (18.8)
T3/4	3 (30.0)	441 (45.4)
Not assessed	4 (40.0)	347 (35.7)
Length of neoadjuvant treatment		
9–18 ws	4 (40.0)	547 (56.3)
18–23 ws	1 (10.0)	44 (4.5)
24 ws	3 (30.0)	141 (14.5)
Not assessed	2 (20.0)	239 (24.6)
HER2 status		
Negative	4 (40.0)	352 (36.3)
Negative/Positive	5 (50.0)	351/29 (36.1/3.0)^b^
Not assessed	1 (10.0)	239 (24.6)
Research contents		
NET vs NCT	6 (60.0)	768 (79.1)
NECT vs monotherapy	4 (40.0)	203 (20.9)

*Abbreviations*: *NET* Neoadjuvant endocrine therapy, *NCT* Neoadjuvant chemotherapy, *NECT* Neoadjuvant chemoendocrine therapy, *RECIST v1.1* Response Evaluation Criteria in Solid Tumours, version 1.1, *WHO* World Health Organization

^a^The median value is calculated by available data

^b^These data before and after the semicolon respectively represent the number of HER2-positive disease and HER2-negative disease patients

**Table 2:** The treatment regimen of neoadjuvant therapy

Study	Year	NET	NCT	NCET
Chae [24]	2016	Letrozole qd	FEC, a switch to docetaxel if PD or SD	
Wright [25]	2015	AIs or tamoxifen qd	PAT or AT	
Palmieri [26]	2014	Letrozole qd	FE100C or FE75C, a switch to docetaxel if PD or SD	
Semiglazov [27]	2007	Exemestane or anastrozole qd	Doxorubicin + paclitaxel	
Marcus [28]	2013	AIs or tamoxifen qd	Anthracycline-based or non-anthracycline-based	
Ellis [29]	2017	AIs qd^a^	Anthracycline-based or non-anthracycline-based	
Nakayama [30]	2018	Anastrozole qd		Anastrozole qd + UFT
Sato [31]	2018	Exemestane qd		Exemestane qd + cyclophosphamide
Sugiu [32]	2015		FEC-T	Exemestane qd + EFC-T
Mohammad [33]	2012		FAC	Letrozole qd + FAC

Explanation of regimen: *FEC*, 5-fluorouracil, epirubicin, and cyclophosphamide; *FE100C*, 5-fluorouracil 500 mg/m^2^, cyclophosphamide 500 mg/m^2^, epirubicin 100 mg/m^2^; *FE75C*, 5-fluorouracil 600 mg/m^2^, cyclophosphamide 600 mg/m^2^, epirubicin 75 mg/m^2^; *UFT*, tegafur/uracil combination in 1:4 M ratio; 270 mg/m^2^/day in two divided doses; *FEC-T*, 80 mg/m^2^ of paclitaxel followed by a combination of fluorouracil 500 mg/m^2^, epirubicin 100 mg/m^2^ and cyclophosphamide 500 mg/m^2^; *FAC*, 5-Fluorouracil 600 mg/m^2^, doxorubicin 60 mg/m^2^ and cyclophosphamide 600 mg/m^2^

*Abbreviations*: *NET* Neoadjuvant endocrine therapy, *NCT* Neoadjuvant chemotherapy, *NCET* Neoadjuvant chemoendocrine therapy, *AIs* Aromatase inhibitors, *PD* Progressive disease, *SD* Stable disease

^a^These aromatase inhibitors include letrozole, anastrozole and exemestane

### NET vs NCT

**Fig. 2:**
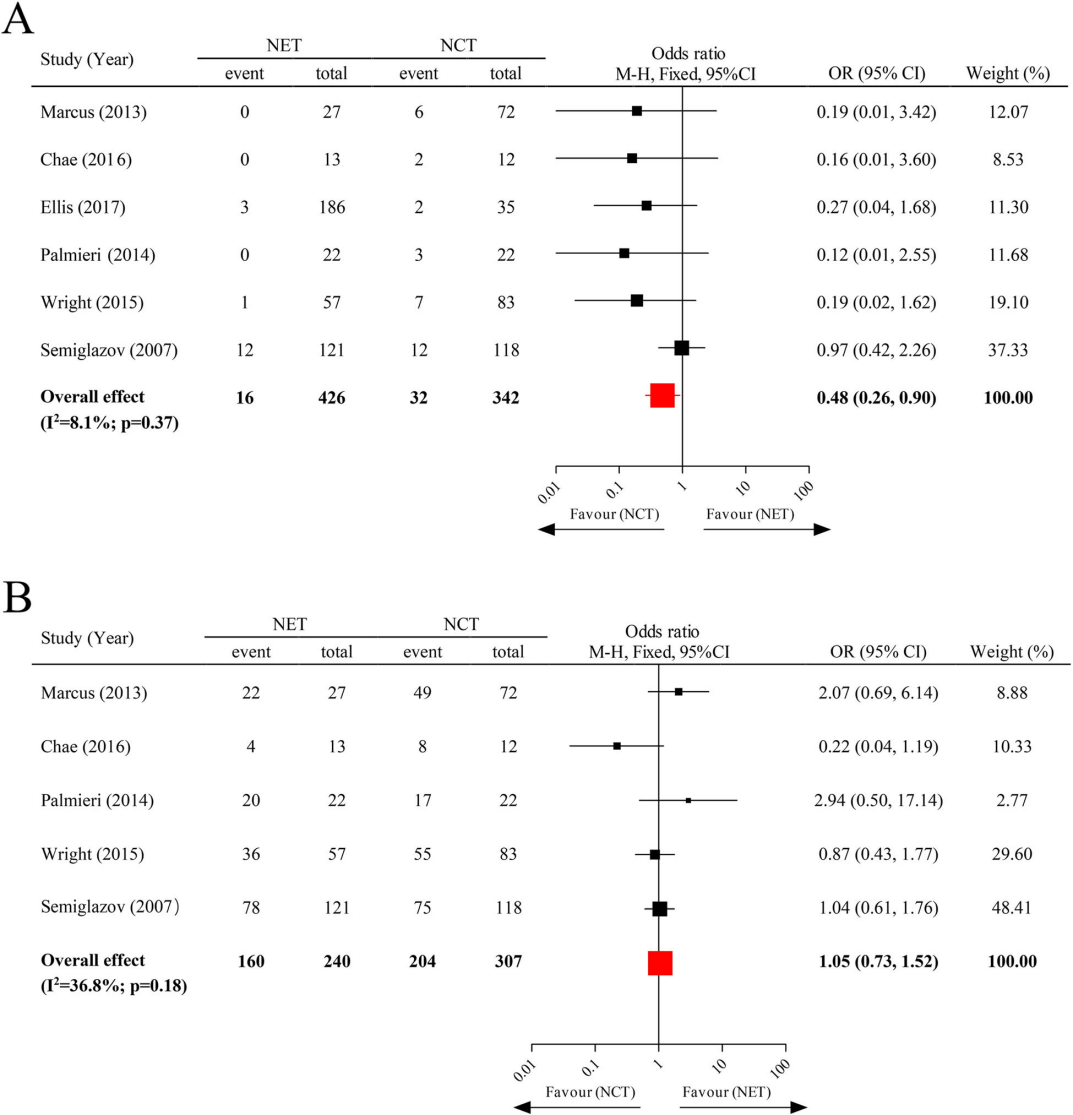
The pCR rate and ORR of neoadjuvant endocrine therapy vs neoadjuvant chemotherapy. Abbreviations: NET, neoadjuvant endocrine therapy; NCT, neoadjuvant chemotherapy

Six clinical trials were involved in analysing the pCR of NET vs NCT, with a total number of 768 subjects. Of these, the American College of Surgeons Oncology Group (ACOSOG) Z1031 trial [20] did not investigate the ORR of breast tumour after neoadjuvant therapy, thereby giving rise to only 5 articles for analysis of the ORR of NET vs NCT. The pooled results indicated that the pCR rate of postmenopausal, HR-positive breast tumour patients after receiving NET was significantly lower than those that underwent NCT (pooled OR = 0.48; 95% CI, 0.26–0.90), whereas no significant difference of ORR existed between those that underwent these two treatment paradigms (pooled OR = 1.05; 95% CI, 0.73–1.52) (Fig. 2).

### NCET vs Monotherapy of NET or NCT

**Fig. 3:**
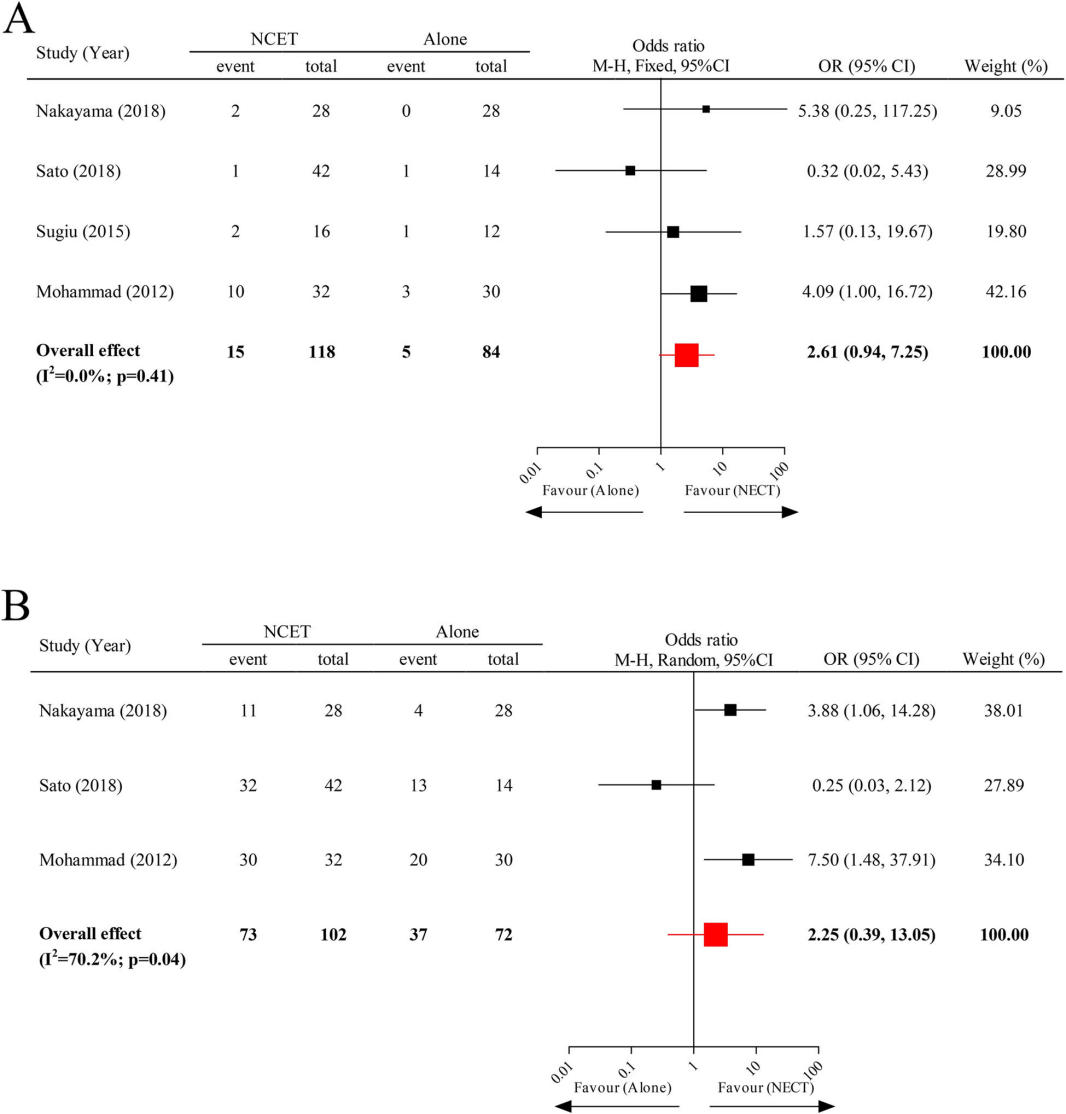
The pCR rate and ORR of neoadjuvant endocrine therapy vs neoadjuvant chemoendocrine therapy. Abbreviations: NET, neoadjuvant endocrine therapy; NCET, neoadjuvant chemoendocrine therapy

To investigate this comparison, three RCTs and one retrospective study (total sample size of 203) were ultimately collected. Irrespective of HR-positive breast cancer patients receiving combined treatment of NCET or monotherapy of NET or NCT, the differences in pCR rate and ORR between them were not statistically significant (pooled OR = 2.61; 95% CI, 0.94–7.25; and 2.25; 95% CI, 0.39–13.05; respectively) (Fig. 3).

### Risk of bias in included studies

**Fig. 4:**
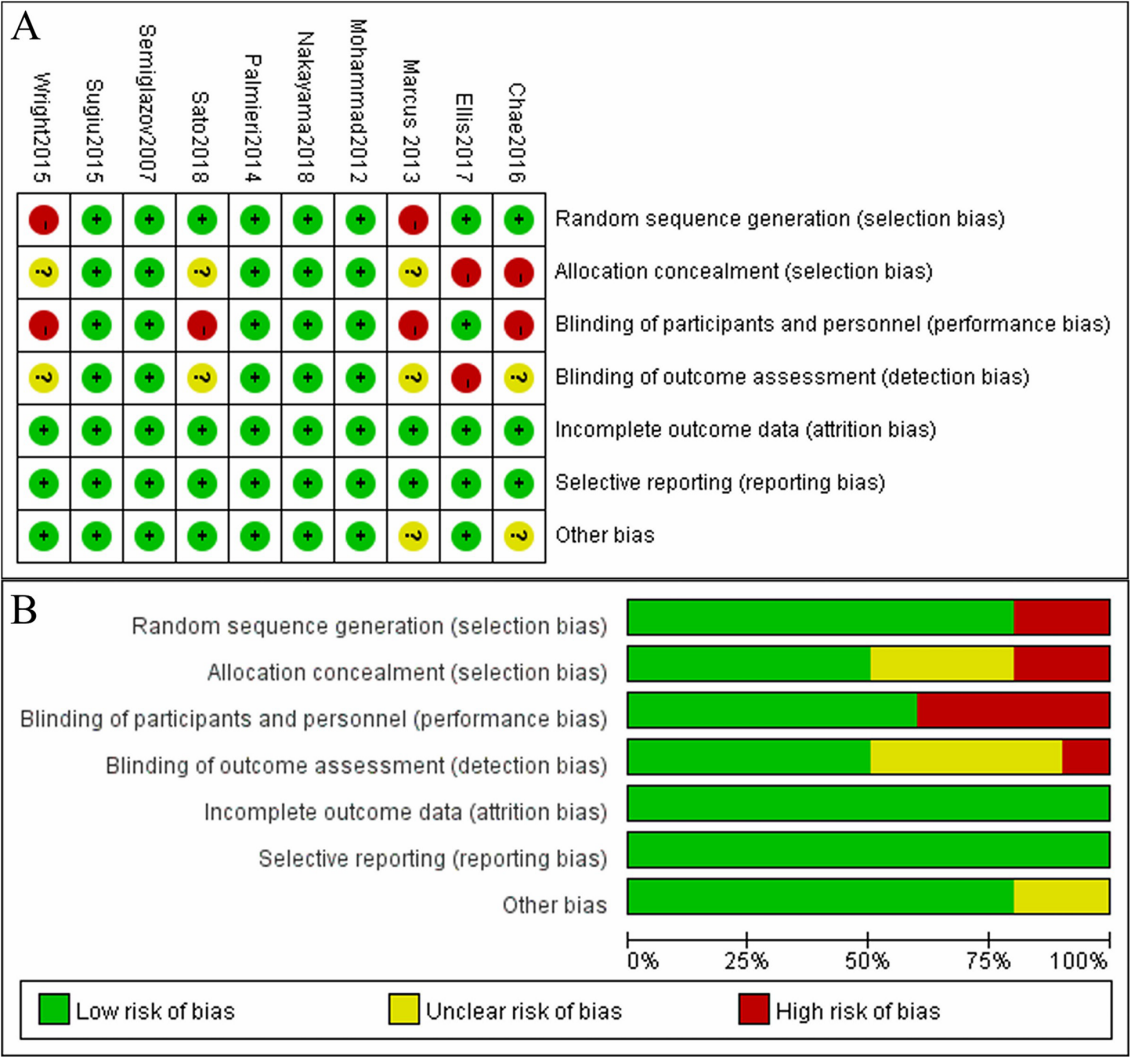
The judgements of risk of bias summary and risk of bias graph

Figure 4A and B showed the judgements of risk of bias summary and risk of bias graph, respectively, of the analysed studies for each “Risk of bias” domain. The detailed risk of bias assessments was provided in Additional file 1: Table S2, *page 2*.

### Heterogeneity and publication bias

Most of the studies in this meta-analysis did not appear to contain heterogeneity, as their Heterogeneity Chi^2^ tests were not statistically significant (*p* > 0.1); thus, the fixed-effect model was adopted to calculate the value of pooled OR. There was an exception, which was the evaluation of the ORR between NCET with NET or NCT alone, in which the value of that was computed by the random-effect model. Whilst its publication bias was not significant according to the results of Egger’s test (*p* = 0.452) (Additional file 1: Table S3, page 3).

## Discussion

The popularity of neoadjuvant therapy in the treatment of breast cancer greatly increases the proportion of breast-conserving surgeries, prolongs the long-term survival of patients and improves their quality of life. With an increasing body of clinical studies proving that endocrine therapy drugs, such as tamoxifen and, subsequently, emergent fulvestrant and aromatase inhibitors (AIs), have preeminent curative effects on HR-positive breast cancers, a consensus has been reached that endocrinotherapy is preferred to chemotherapy for such patients because the former treatment is less toxic and easier tolerated than the latter one. Otherwise, for patients with endocrine drug-resistance, rapid tumour progression or visceral crisis NCT, which has high efficiency in tumour regression, is selected because NET takes effect slowly, commonly needing 3–4 months to achieve observable tumour shrinkage [34]. However, it is not recommended to integrate chemotherapy into endocrine therapy to treat HR-positive breast tumours. Our results demonstrated that postmenopausal HR-positive breast cancer patients undergoing NCT were prone to obtain pCR compared to those that received NET, but there was no difference in ORR between the two treatment paradigms, and NCET was not more efficient than NET or NCT alone in achieving pCR or tumour clinical response.

Ki67 is the most commonly used biomarker in endocrine therapy trials and the tool to differentiate HR-positive breast cancer into Luminal A and Luminal B molecular subtype diseases, in parallel, which can also be utilized to predict tumor response and long-term outcomes after endocrine therapy in neoadjuvant setting [20, 35, 36]. The ACOSOG Z1031A trial [20] registered 245 postmenopausal HR-positive breast cancer patients who accepted the breast biopsy after 2 weeks of NET to ascertain Ki67 index. Of those patients, 35 switched to 4 weeks of NCT because of Ki67>10% who experienced high a pCR rate of 5.7% and were more likely to undergo mastectomy and axillary lymph node dissection than those patients continuously received 4 weeks of NET, indicating that chemotherapy efficacy was lower than expected in HR-positive breast cancers resistant to NET. Nevertheless, a randomized controlled trial of Palmieri et al. who did not distinguish HR-positive breast tumour patients in light of the Ki67 index found a similar breast-conserving surgery rate between those patients undergoing NET and NCT [21]. Overall, the biomarker Ki67 may impact on the decision of extent of breast surgery.

Earlier studies reported the promising prognosis in HR-positive breast cancer patients who underwent endocrine therapy with tamoxifen [37–39]. Currently, fulvestrant is the only and so-called “pure anti-oestrogen” oestrogen receptor downregulator without any agonist effects. In advanced HR-positive breast tumour patients, fulvestrant is often used in combination with a cyclin-dependent kinase 4 and 6 (CDK4/6) inhibitor, which can significantly prolong progression-free survival compared to fulvestrant alone (9.2 months vs 3.5 months; *P* = 0.007) [40]; in patients sensitive to endocrine therapy, this combination therapy also brings about longer OS than fulvestrant monotherapy (39.7 months vs 29.7 months; Hazard ratio, 0.92; 95% CI, 0.55–0.94) [41]. In postmenopausal women, the most oestrogen is produced via androstenedione synthesized from adrenal gland enters into peripheral adipose tissue that is converted to oestrogen by 17-hydroxysteroid dehydrogenase [42]. AIs can inhibit the activity of 17-hydroxysteroid dehydrogenase and result in preventing the conversion of androstenedione to oestrogen. A large number of clinical studies indicated that the usage of the third generation AIs in postmenopausal HR-positive breast cancer patients outperformed tamoxifen in favourable long-term survival, improved tumour response and increased the breast-conserving surgery rate [43–47].

Although there have been a large number of studies concluding the increased clinical response rate of tumours in the combined strategy of chemotherapy and endocrinotherapy compared to chemotherapy alone to treat advanced HR-positive breast tumours [48–50], there are a paucity of clinical trials comparing the clinical utility of the combination to endocrinotherapy or chemotherapy in a neoadjuvant setting. Of these, most indicate no differences of pCR rate and ORR between postmenopausal HR-positive breast cancer women who undergo NCET and monotherapy of NET or NCT, which is consistent with our results. One probable explanation is that endocrine therapy works through a cytostatic mechanism to prevent cell division and downsize tumour volume; in contrast, chemotherapeutic medicines will be more effective when the cycling of cells in patients is more rapid [51]. Consequently, in theory, the coexistence of chemotherapy and endocrine agents in NCET may give rise to an antagonistic effect that does not reinforce the efficacy of this combined therapy. This conclusion is consolidated by a systematic review of 20 studies with 3490 unique patients that NET as monotherapy manifested a similar clinical response rate but significantly attenuated toxicity when compared with NCET [52].

Justifiably, the results of some clinical trials showed that the safety of NCET was less than that of NET alone [11, 14], especially in high-grade (Grade ≥ 3) adverse events (AEs) including increased aspartate transaminase, increased alanine aminotransferase, increased γ-glutamyl transpeptidase, back pain, and hypertriglyceridemia, as well as arthralgia but without treatment-related death. However, compared with NCT arm, the high-grade AEs in NCET arm was no significant difference [13].

Admittedly, this meta-analysis had some limitations. First, the inclusion criteria stipulated that only English publications were eligible, which might lead to selection bias and publication bias; undeniably, there was publication bias in comparing the pCR rate between NET and NCT (Additional file 1: Table S2, *page 2*). Second, since few clinical trials evaluated the comparison of tumour response rate between postmenopausal HR-positive breast tumours after combination of NCET with monotherapy of NET or NCT, only 4 valid studies were involved to analyse this comparison, and considerable heterogeneity existed in the meta-analysis comparing ORR between both paradigms, which might cause resultant bias. Finally, there might be clinical heterogeneity by virtue of the differences in HER2 status of patients, assessment criteria for tumour response, and treatment regimen in analysed studies.

This is the first study that systematically investigates the tumour response rate in postmenopausal HR-positive breast tumours after NET, NCT or NCET, finding some clinically promising outcomes. Our results further consolidate the consensus that NET is the preferred alternative for the treatment of these patients, with the exception of those that needs rapid reduction of tumour volume or who are resistant to endocrinotherapy.

## Conclusion

Postmenopausal HR-positive breast cancer patients may benefit more tumour response from NCT than NET, but may be devoid of the improved prognostic outcomes from NCET when compared to NET or NCT alone.

## Supplementary information

**Additional file 1: Table S1.** Study quality of eligible randomized controlled trials. **Table S2.** The detailed risk of bias assessments. **Table S3.** The publication bias by Egger’s test.

## Data Availability

The datasets used and/or analyzed during the current study are available from the corresponding author on reasonable request.

## Abbreviations

ACOSOG: American College of Surgeons Oncology Group
AEs: Adverse events
AIs: Aromatase inhibitors
CDK4/6: Cyclin-dependent kinase 4 and 6
CIs: Confidence intervals
DFS: Disease-free survival
HER2: Human epidermal growth factor receptor 2
HR: Hormone receptor
NCET: Neoadjuvant chemoendocrine therapy
NCT: Neoadjuvant chemotherapy
NET: Neoadjuvant endocrinotherapy
NSABP: National Surgical Adjuvant Breast and Bowel Project
OR: Odds ratio
ORR: Overall response rate
OS: Overall survival
pCR: Pathological complete response
