# Supplementary material for: The tumour response of postmenopausal hormone receptor-positive breast cancers undergoing different types of neoadjuvant therapy: a meta-analysis
Source: BMC Womens Health. 2020 Jan 31;20:17. doi: 10.1186/s12905-020-0879-y (PMC6993383; doi:10.1186/s12905-020-0879-y)
Supplement: Supplementary file 1 — Additional file 1: Table S1. Study quality of eligible randomized controlled trials. Table S2. The detailed risk of bias assessments. Table S3. The publication bias by Egger’s test. [file 12905_2020_879_MOESM1_ESM.docx]

| ***First author*** | ***Publication year*** | ***Study  described as  randomized?*** | ***Randomization  method  described and  appropriate?*** | ***Study  described as  double*** ***blind?*** | ***Method of  double blinding  described and  appropriate?*** | ***Description of  withdrawals  and dropouts?*** | ***Jadad  Score  (0-5)*** |
| --- | --- | --- | --- | --- | --- | --- | --- |
| Chae | 2016 | Yes | Yes | Not reported | Not reported | Yes | 3 |
| Palmieri | 2014 | Yes | Yes | Not reported | Not reported | Yes | 3 |
| Semiglazov | 2007 | Yes | Yes | Not reported | Not reported | Yes | 3 |
| Nakayama | 2018 | Yes | Yes | Not reported | Not reported | Yes | 3 |
| Ellis | 2017 | Yes | Yes | Not reported | Not reported | Yes | 3 |
| Sugiu | 2015 | Yes | Yes | Not reported | Not reported | Yes | 3 |
| Mohammad | 2012 | Yes | Yes | Not reported | Not reported | Yes | 3 |

**Table S1. Study quality of eligible randomized controlled trials.**

|  | **Risk of bias summary** | **Low risk of bias (%)** |
| --- | --- | --- |
| **Random sequence generation** | Seven studies are described as randomized. One study describes the method of random sequence generation adequately, i.e. with low risk of bias. The remained two studies are at high risk of bias. | 75-100 |
| **Allocation concealment** | Five studies describe the method of allocation concealment adequately, i.e. with low risk of bias. Three studies do not provide sufficient information to accurately assess the method of allocation concealment, therefore are at unclear risk of bias. The remained two studies are at high risk of bias. | 50 |
| **Blinding of participants and personnel** | Six studies describe the method of blinding of participants and personnel adequately, i.e. with low risk of bias. Other four studies are at high risk of bias. | 50-75 |
| **Blinding of outcome assessments** | Fix studies describe the method of blinding of outcomes assessments adequately, i.e. with low risk of bias. Three studies providing insufficient details to judge the method of blinding of outcomes assessments are at unclear risk of bias. Only one study is at high risk of bias. | 50 |
| **Incomplete outcome data** | All studies are generally free of attrition bias. | 100 |
| **Selective reporting** | All studies are generally free of reporting bias. | 100 |
| **Other bias** | Eight studies are free of other bias, but the other two studies are at unclear risk of bias. | 75-100 |

**TableS2. The detailed risk of bias assessments.**

| Analyzed label | p value^*^ |
| --- | --- |
| *pCR* |  |
| NET vs NCT | 0.002 |
| NCET vs Alone | 0.448 |
| *ORR* |  |
| NET vs NCT | 0.956 |
| NCET vs Alone | 0.452 |

**Table S3. The publication bias by Egger’s test.**

*significant level: p<0.05.

Abbreviations: pCR, pathological complete response; ORR, overall response rate; NET, neoadjuvant endocrinotherapy; NCT, neoadjuvant chemotherapy; NCET, neoadjuvant chemoendocrine therapy.
